# Lymphoma of the Cervix

**DOI:** 10.1155/2012/326127

**Published:** 2012-10-04

**Authors:** Juanita Parnis, David J. Camilleri, Darko Babic, James DeGaetano, Charles Savona-Ventura

**Affiliations:** ^1^Department of Surgery, Mater Dei Hospital, Msida MSD 2090, Malta; ^2^Department of Pathology, Mater Dei Hospital, Msida MSD 2090, Malta; ^3^Department of Obstetrics and Gynaecology, Mater Dei Hospital, Msida MSD 2090, Malta

## Abstract

Primary non-Hodgkins lymphoma of the uterine cervix is a very rare diagnosis. A 54-year-old woman presented with a 3-month history of postmenopausal bleeding per vaginum. On examination, a friable, fungating lesion was seen on the cervix. Histology revealed a CD 20 positive high-grade non-Hodgkin's diffuse large B cell lymphoma from cervical biopsies and endometrial curettage. She was diagnosed as stage IE after workup and subsequently treated with six cycles of R-CHOP chemotherapy followed by radiotherapy of the involved field.

## 1. Introduction

Female genital tract lymphomas are rare diseases [1]. They account for 1.5% of extranodal Non-Hodgkin's Lymphomas and less than 0.5% of gynaecological cancers [2]. Cervical involvement in multiorgan disease is more common than primary lymphoma [3]. Non-Hodgkin's Lymphoma (NHL) can involve extranodal sites in about one-third of patients [4]. Common extranodal locations include the gastrointestinal tract and the skin. However, the female reproductive system may also be affected [5]. Most cases of B-cell lymphomas of the uterine cervix are incidentally detected while women are undergoing routine cervical evaluation by Papanicolaou (Pap) cytology smear screening [6]. Because of the rarity of primary gynaecological lymphomas, a standard treatment has not been defined [7]. 

## 2. Case Presentation

A 54-year-old, married, menopausal woman on Selective Serotonin Reuptake Inhibitors (SSRIs), presented to Gynaecology Out-Patient Department with a 3-month history of postmenopausal bleeding. She was menopausal since the age of 50 and had previously undergone a right salpingectomy for an ectopic pregnancy. She had her menarche aged 13 and used to have menorrhagia with irregular periods in the latter part of her reproductive period. She gave birth to one child. 

She had undergone four Dilatation and Curettage's (D&C), the last one being done 10 years previously for irregular periods. The histology then showed benign early luteal phase endometrium and some probable degree of perimenopausal luteal phase dysfunction.

Her current postmenopausal bleeding was managed by Examination Under Anaesthesia (EUA) and D&C. Vulva and vagina were noted as normal but she had a suspicious looking, friable, fungating lesion on the anterolateral part of the cervix. The uterus was anteverted, and mobile. Adnexae were normal. A biopsy from the cervical lesion was taken. No samples were obtained on exploration of the uterine cavity with polyp forceps; however, scanty curettings were obtained on curettage. She had no bleeding at the end of the procedure and was discharged the same day.

**Figure 1:**
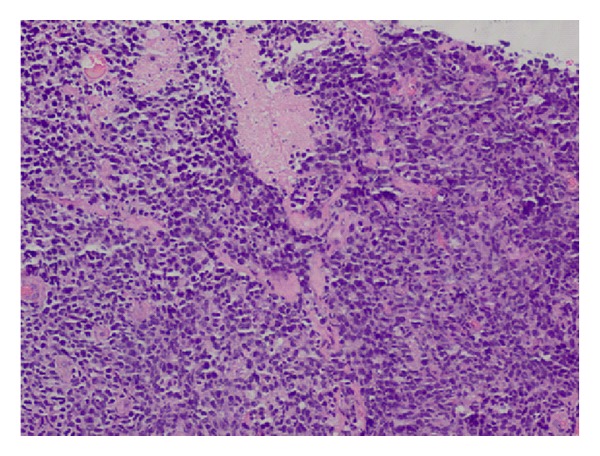
Monotonous neoplastic proliferation of atypical lymphoid cells (H&E 20x).

**Figure 2:**
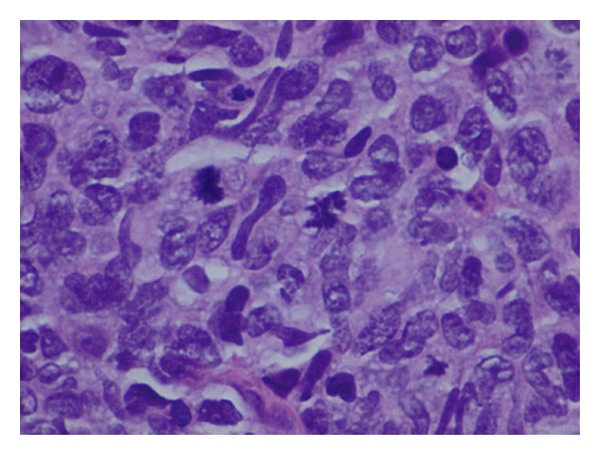
Sheets of large neoplastic lymphoid cells with some cells showing atypical mitoses. Thick capillary vessels and a few eosinophils can also be seen. (H&E 60x).

**Figure 3:**
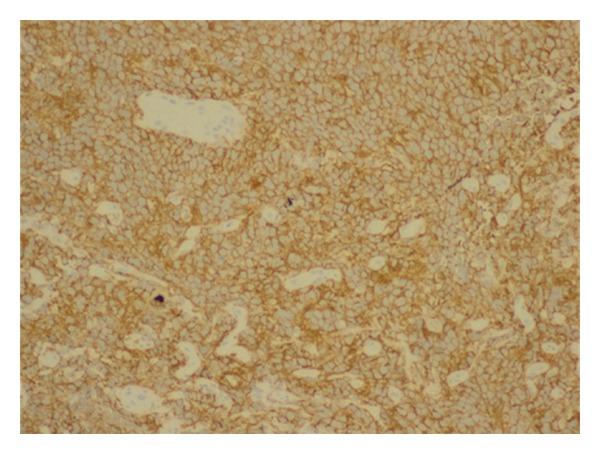
Uniform, strong CD20 positivity of the neoplastic cells (20x).

**Figure 4:**
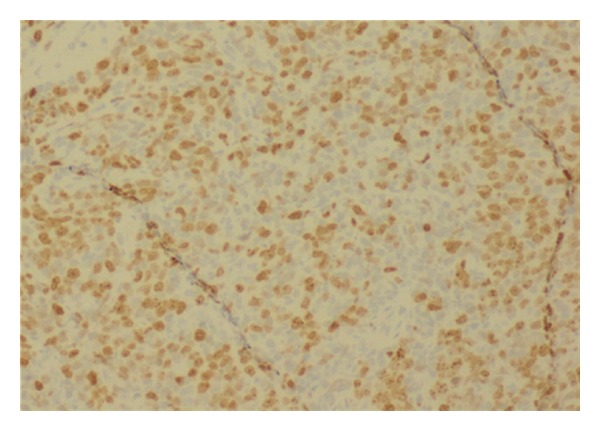
KI67 nuclear positivity of majority of the tumour cells.

The histology report revealed a high-grade non-Hodgkin's diffuse large B-cell lymphoma (DLBCL) from both the cervical biopsies and endometrial curettage specimen. Microscopy on the biopsies from cervix showed multiple fragments consisting of fairly monotonous lymphoid proliferation (Figures 1 and 2) of large LCA and CD20 positive (Figure 3) neoplastic cells, which were also CK, CD3, CD5, Cyclin D1, and Bcl-2 negative. They exhibited high Ki-67 index (Figure 4). Wide areas of necrosis were also present. Endometrial curettage showed few small neoplastic fragments with no remnant endometrium present.

On review by the haematologists in clinic, her blood investigations including a full blood count, erythrocyte sedimentation rate, blood urea and electrolytes, serum creatinine, liver function tests and enzymes, serum calcium and phosphate, C-reactive protein, LDH, and uric acid and serum protein electrophoresis were found to be within normal limits. 

**Figure 5:**
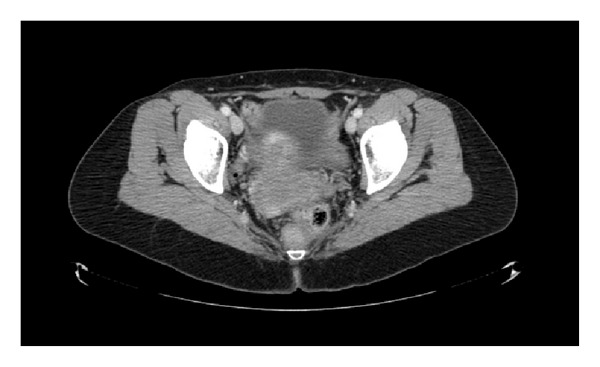
CT scan pretreatment.

A CT thorax and abdomen showed diffuse enlargement of the cervix and lower part of the uterine body with an irregular outline and containing small scattered areas of contrast enhancement. All the lymph node groups seen were normal by radiological criteria (Figure 5).

A staging bone marrow aspirate and trephine were performed. The bone marrow was found to be reactive, showing no neoplastic infiltrate, both microscopically and by flow cytometry. 

Staging was compatible with stage I (E) DLBCL of the cervix uteri. Her R-IPI (Revised International Prognostic Index) score put her in the “low risk” group, with a predicted 4-year progression-free survival of 94% and overall survival of 94% [8]. 

She was scheduled to be treated with R-CHOP (rituximab, cyclophosphamide, adriamycin, vincristine, prednisolone) chemotherapy for four cycles followed by involved field radiotherapy. 

Treatment was complicated by sudden right arm weakness, though CT and MRI of the brain and neck showed no evidence of CVA or CNS lymphoma. The symptoms were thought to be possibly manifestations of a vincristine-induced neuropathy and they ameliorated with omission of the offending drug.

**Figure 6:**
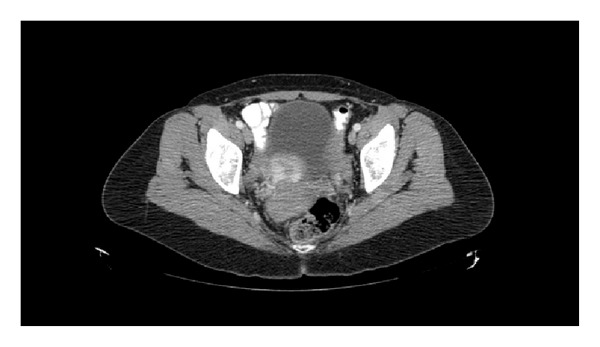
CT scan post-treatment

After 4 cycles of R-CHOP, a restaging CT was performed and this revealed a decrease in size of the cervical mass, however, to less than 50%, which is the minimum reduction in size required to call it a partial remission (Figure 6). 

Repeat biopsies of the residual mass were performed and came back as negative. This was reinforced by a PET-CT which was reported as negative too.

A further 2 Cycles of R-CHOP were administered, whilst the biopsies and PET were organized and she was then referred for radiotherapy.

She received 35 G in 20 fractions over 4 weeks to the cervix, uterus, adnexae, and iliac as well as pelvic and obturator nodes. Radiotherapy was uneventful, bar mild proctitis which resolved.

The patient has now fully recovered and is being followed up in the Haematology Out-Patient Department, one year and five months after diagnosis. 

## 3. Discussion 

Female genital tract lymphomas are rare, accounting for 1.5% of extranodal non-Hodgkin's lymphomas and <0.5% of gynaecological cancers [2]. The median age at presentation is 40 years. 

Patients most often present with one or more episodes of vaginal bleeding (70%), perineal discomfort (40%), and persistent vaginal discharge (20%) [4].

The actual diagnosis may be difficult to reach and usually requires slide review by haematohistopathologists.

A distinction between two patient groups needs to be made in this context. There is one group, presenting with symptoms and signs, but no obvious clinical/radiological cervical mass, described as having “lymphoma-like lesions” (LLL), that may be associated with chronic Chlamydial, EBV, or Papilloma Virus Infection. This group of patients present certain characteristics, such as the lack of a clinical/radiological mass, lack of invasiveness, cellular pleomorphism, and a residual population of normal T-Cells admixed with the neoplastic cells. It has been found that a “watch and wait” approach with no treatment is appropriate for such a group of patients, since these LLLs resolve spontaneously [9, 10].

The other group of patients, into which our patient falls, is that where symptoms and signs are associated with a clinical and radiological mass. This group has to be offered treatment, though adequate therapy for this rare condition has never been standardized. 

In reported cases, the tumour has been managed with chemotherapy and surgery. This approach demonstrated a long disease-free followup of up to 10 years for individual patients. In these reports, the management approach included six cycles of CHOP chemotherapy followed by surgery. 

The use of chemotherapy and radiation was reported by Stroh et al. who reported 16 cases of lymphomas of the cervix of which 12 had received radiation. In this series, 90% of patients with low risk factors were disease-free at 5 years [7]. More recently, case reports, such as that by Heredia et al. demonstrate the use of combination of CHOP × 3 plus involved field radiotherapy as therapy for this malignancy [11]. One must conclude that combined modality treatment with CHOP-based chemotherapy and radiation is the best treatment for DLBCL of the cervix.

## 4. Conclusion

This presentation of cervical DLBCL highlights how important it is to have an open mind when working up patients presenting with common symptoms in gynaecology and also reviews the diagnostic and therapeutic differences between LLL and DLBCL, and the different management approaches for these rare conditions.
